# Prevalence and predictors of germline *CDKN2A* mutations for melanoma cases from Australia, Spain and the United Kingdom

**DOI:** 10.1186/1897-4287-12-20

**Published:** 2014-11-20

**Authors:** Mark Harland, Anne E Cust, Celia Badenas, Yu-Mei Chang, Elizabeth A Holland, Paula Aguilera, Joanne F Aitken, Bruce K Armstrong, Jennifer H Barrett, Cristina Carrera, May Chan, Joanne Gascoyne, Graham G Giles, Chantelle Agha-Hamilton, John L Hopper, Mark A Jenkins, Peter A Kanetsky, Richard F Kefford, Isabel Kolm, Johanna Lowery, Josep Malvehy, Zighereda Ogbah, Joan-Anton Puig-Butille, Jordi Orihuela-Segalés, Juliette A Randerson-Moor, Helen Schmid, Claire F Taylor, Linda Whitaker, D Timothy Bishop, Graham J Mann, Julia A Newton-Bishop, Susana Puig

**Affiliations:** Section of Epidemiology and Biostatistics, Leeds Institute of Cancer and Pathology (LICAP), University of Leeds, Leeds, UK; Cancer Epidemiology and Services Research (CESR), Sydney School of Public Health, Sydney Medical School, The University of Sydney, Sydney, Australia; Dermatology Department and Biochemistry and Molecular Genetics Department, Melanoma Unit, Hospital Clinic, Instituto de Investigaciones Biomédicas August Pi I Sunyer (IDIBAPS), Barcelona, Spain; Centro Investigación Biomédica en Enfermedades Raras (CIBERER), Instituto de Salud Carlos III (ISCIII), Barcelona, Spain; Westmead Institute for Cancer Research and Melanoma Institute, Australia, University of Sydney at Westmead Millennium Institute, Sydney, Australia; Viertel Centre for Research in Cancer Control, The Cancer Council Queensland, Spring Hill, Brisbane, Australia; Centre for Epidemiology & Biostatistics, School of Population Health, University of Melbourne, Melbourne, Australia; Cancer Epidemiology Centre, Cancer Council Victoria, Melbourne, Australia; Department of Cancer Epidemiology, H. Lee Moffitt Cancer Center & Research Institute, Tampa, FL USA; Genomics Facility, Leeds Cancer Research UK Centre, University of Leeds, Leeds, UK; Medicina Laboral, Lafarge Cementos y Finanzauto, S.A., Barcelona, Spain

**Keywords:** Family history, Multiple primaries, Population-based, Melanoma, *CDKN2A*

## Abstract

**Background:**

Mutations in the *CDKN2A* and *CDK4* genes predispose to melanoma. From three case-control studies of cutaneous melanoma, we estimated the prevalence and predictors of these mutations for people from regions with widely differing latitudes and melanoma incidence.

**Methods:**

Population-based cases and controls from the United Kingdom (1586 cases, 499 controls) and Australia (596 early-onset cases, 476 controls), and a hospital-based series from Spain (747 cases, 109 controls), were screened for variants in all exons of *CDKN2A* and the p16INK4A binding domain of *CDK4*.

**Results:**

The prevalence of mutations for people with melanoma was similar across regions: 2.3%, 2.5% and 2.0% for Australia, Spain and the United Kingdom respectively. The strongest predictors of carrying a mutation were having multiple primaries (odds ratio (OR) = 5.4, 95% confidence interval (CI: 2.5, 11.6) for 2 primaries and OR = 32.4 (95% CI: 14.7, 71.2) for 3 or more compared with 1 primary only); and family history (OR = 3.8; 95% CI:1.89, 7.5) for 1 affected first- or second-degree relative and OR = 23.2 (95% CI: 11.3, 47.6) for 2 or more compared with no affected relatives). Only 1.1% of melanoma cases with neither a family history nor multiple primaries had mutations.

**Conclusions:**

There is a low probability (<2%) of detecting a germline *CDKN2A* mutation in people with melanoma except for those with a strong family history of melanoma (≥2 affected relatives, 25%), three or more primary melanomas (29%), or more than one primary melanoma who also have other affected relatives (27%).

**Electronic supplementary material:**

The online version of this article (doi:10.1186/1897-4287-12-20) contains supplementary material, which is available to authorized users.

## Background

Cutaneous malignant melanoma is responsible for most skin cancer mortality worldwide and continues to increase in incidence in the most susceptible populations [1, 2].

The principal high-penetrance susceptibility gene identified as pathogenic to date is the *CDKN2A* locus on chromosome 9 [3]. Additionally, a small number of melanoma pedigrees have been found to carry mutations of the *CDK4* gene, at the binding site for the *CDKN2A* gene product [4, 5]. For members of families with multiple cases of melanoma, the presence of relatives with multiple primary melanoma (MPM), pancreatic cancer, and early age at onset predict the presence of germline *CDKN2A* mutations [6–9]. The predictors of mutation status for melanoma cases who have not been selected for family history or MPM are much less well understood.

There are only limited data available on the prevalence of *CDKN2A* and *CDK4* mutations outside the context of familial melanoma. Some small, early studies have reported very high prevalence, especially for cases of MPM [10]. A population-based Norwegian study of MPM cases reported a mutation prevalence of 21.0% for familial cases and 3.2% for the non-familial cases [11]. A large, international, population-based study recruiting from a range of latitudes reported a mutation prevalence of 1.2% for cases with a single primary melanoma and 2.9% for MPM cases [12]. Previous studies have suggested that in Mediterranean countries and Latin American countries with a relatively low incidence of melanoma, the proportion of cases in the population with germline mutations might be higher [13, 14]. Early age at onset in the absence of a family history might only be a weak predictor of *CDKN2A* mutations, which notably are rare in childhood and adolescent melanoma (1.4% of cases in one study) [15].

We report here the prevalence and predictors of carrying a pathogenic *CDKN2A* or *CDK4* exon 2 mutation for three populations, UK, Spain and Australia, with different phenotypes (Mediterranean compared with North European), living at very different latitudes (from 37° south to 53° north), and for cases with a wide range of age at diagnosis and family history. We have previously reported that the penetrance of *CDKN2A* mutations in population-ascertained families is similar in the UK and Australia; mutation carriers appeared to have the same cumulative risk of melanoma irrespective of ambient UV irradiance [16]. We hypothesized that the prevalence of germline mutations would be: 1) higher for melanoma cases living in a country with lower levels of sun exposure (UK) than for melanoma cases of a similar ethnicity living in a country with much higher levels of sun exposure (Australia); 2) higher for cases with MPM and a family history than for those without; and 3) higher for early-onset cases than for late-onset cases. We also report the prevalence of non-coding variants (p.A148T and the 3′UTR variants) of *CDKN2A*.

## Methods

### Samples and subjects

The frequency of germline *CDKN2A* and *CDK4* mutations was investigated using population-based series of melanoma cases and population-ascertained controls from Leeds, United Kingdom (previously described [17]) and Australian cities (Brisbane, Sydney and Melbourne) in the Australian Melanoma Family Study [18]. A third cohort of cases and controls was ascertained through the Melanoma Unit in the Hospital Clinic of Barcelona, Spain.

In the UK, population-ascertained incident pathologically confirmed melanoma cases have been recruited to a case-control study since 2000 in a geographically defined area of Yorkshire and the Northern region of the UK (67% case participation). Only cases with invasive lesions were recruited. 2085 male and female people (aged between 18 and 82 years) were diagnosed in the period from September 2000 to September 2011. Here we report on the first 1586 cases for which germline DNA was screened. The cases were identified through clinicians, pathology registers and the cancer registry to ensure maximal ascertainment. Between September 2000 and June 2003 all people with invasive melanoma were invited to participate. From July 2003 to September 2011, cases with Breslow thickness less than 0.75 mm were not invited in order to enrich the cohort to observe clinical outcomes. 513 population-ascertained controls were identified from the cases’ family doctors; 55% of controls who expressed an interest in participation did so. The first 499 of the UK population-based controls were screened in this study. Cases and controls were asked about family history of melanoma and previous melanomas. Analyses of family history for this manuscript were based on self-report.

In Australia, 629 people with histopathologically-confirmed first primary invasive melanoma diagnosed at ages 18-39 years in the greater urban areas of Brisbane, Sydney and Melbourne were ascertained between 1st July 2000 and 31st Dec 2002, together with their first- and second-degree relatives. Those with a previous invasive melanoma were not eligible, but 15 who presented with synchronous primary melanomas or a previous *in situ* primary melanoma remained eligible; participation was 54% of those eligible and 76% of those contactable. Population-based, age-city-sex-matched controls (n = 240) were ascertained from the electoral roll for each state (participation was 23% of those eligible and 42% of those contactable), and 295 spouse/friend controls (80% of those nominated participated and there was a spouse/friend control for 47% of cases). The population and spouse/friend control groups were similar in demographic detail, ancestry and prevalence of a reported family history of melanoma, and so were combined for this analysis. All those with blood samples (n = 596 cases and 476 controls) were screened for mutations.

The recruits from Barcelona, Spain, were identified from a clinic-based series. The Melanoma Unit at the Hospital Clinic in Barcelona manages 36% of all melanoma cases presenting to hospitals in Catalonia; a population of 7 million inhabitants with ~710 new cases of melanoma per year in the last 5 years (registry of the Xarxa Catalana de Melanoma). All cases with a diagnosis of at least one melanoma and with available DNA were eligible. Overall participation was 64%. In 2002, during a period of 2 months, all workers presenting for annual health review at Lafarge Cementos and Finanzauto, S.A (a local construction supply company) were invited to participate as controls; participation was 68%. A total of 747 Spanish cases recruited from June 1999 to January 2004 and 109 controls were screened for mutations.

Ethics committee approval for these studies was obtained from all relevant multiregional and local ethics committees in each country.

### Mutation screening

Mutation screening of *CDKN2A* exons 1α, 1β, 2, 3 and *CDK4* exon 2 was carried out at the core centre of each study: bidirectional DNA sequencing (*CDKN2A* exons 1α, 1β, 3 and *CDK4* exon 2) and denaturing high performance liquid chromatography (DHPLC) (*CDKN2A* exon 2 only) at the Sydney core of the Australian Melanoma Family Study; single strand conformation polymorphism (SSCP) analysis at Barcelona; and either DHPLC or high resolution melting curve analysis (HRMCA) at Leeds. Primers, protocols and reaction conditions were as previously described for SSCP [13]; DHPLC [19]; and HRMCA [20, 21]. The equivalence of these methods for identifying mutations has previously been confirmed [19].

### Sequencing analysis of SSCP, DHPLC and HRMCA ‘positive’ samples

All amplicons that displayed an aberrant SSCP, DHPLC or HRMCA profile were bi-directionally sequenced as previously described [19] to identify the underlying nucleotide change.

### Assignment of significance of status to variants

For the purposes of this study the observed variants were classified as ‘pathogenic’, that is, they are presumed to predispose to melanoma, or as ‘non-pathogenic’. Variants were classified according to a flow chart (Additional file 1), using the following criteria: having functional evidence of impaired protein function; predicted to result in protein truncation (via nonsense mutation, frameshift, or altered splicing); previously reported co-segregation with disease in large melanoma families; or, if these data were not available, prediction of a strong deleterious effect on *CDKN2A* protein by bioinformatic criteria based on SIFT (http://sift.jcvi.org/), Polyphen (http://genetics.bwh.harvard.edu/pph/) or pMut (http://mmb2.pcb.ub.es:8080/PMut/). The common *CDKN2A* variant p.A148T (rs3731249) and the 3′ UTR variants c.*29C > G (rs11515) and c.*69C > T (rs3088440) were classified as pathogenic ‘low penetrance’ variants, based on previous investigations into their possible association with melanoma [22–26], and were analyzed separately.

### Statistical methods

Cochran-Mantel-Haenszel tests were used to determine whether the prevalence of mutation differed by melanoma status, family history of melanoma, having multiple primary of melanomas and country. Wilcoxon signed ranks tests were used to determine whether the prevalence of pathogenic mutations differed by tumour thickness. Difference in means of quantitative factors, e.g. age, were assessed by Student’s T Test. Distributions of qualitative factors were compared across groups using Fisher’s Exact Test.

Unconditional logistic regression was used to assess the impact of age at diagnosis, family history of melanoma (in first- and second degree relatives: 0, 1, 2 or more relatives) and the presence of multiple primaries (1, 2, 3 or more primaries) on the odds of carrying a pathogenic *CDKN2A* mutation. Results are reported as odds ratios (ORs) and 95% confidence intervals (CIs). We also estimated the crude ORs and 95% CIs for melanoma associated with carrying the low penetrance variants p.A148T, c.*29C > G and c.*69C > T. All analyses were carried out using SAS version 9.1 (SAS Institute Inc. Cary, NC, USA), or Stata.

We conducted goodness of fit tests using a multivariate model containing family history, presence of multiple primaries, age and sex, to examine how well this model predicted the number of observed mutation carriers in our study samples from each country.

## Results

### Analysis of pathogenic *CDKN2A*mutations

The numbers of cases and controls screened at each centre, age at diagnosis (cases) or recruitment (controls), the proportion of cases with a family history of melanoma, and the presence of MPM are shown in Table 1. Information on family history and MPM is based on information available at the time of recruitment.

**Table 1:** **Descriptive characteristics of individuals recruited to studies**

Country		Australia		Spain		UK	
Case/Control		Case	Control	Case	Control	Case	Control
Number screened		596	476	747	109	1586	499
Age at diagnosis/recruitment^1^	< 40 (%)	596 (100)	377 (79)	232 (31)	18 (17)	289 (18)	61 (12)
	≥ 40 (%)	0	99 (21)	508 (69)	91 (83)	1297 (82)	438 (88)
Affected relatives^1,2^	0 (%)	500 (83.9)	-	690 (93.2)	-	1470 (92.7)	-
	1 (%)	76 (12.8)	-	34 (4.6)	-	101 (6.3)	-
	2+ (%)	20 (3.4)	-	16 (2.2)	-	15 (0.9)	-
Number of melanoma primaries^1,2^	1	581 (97.5)	-	639 (86.4)	-	1495 (94.3)	-
	2	14 (2.4)	-	80 (10.8)	-	73 (4.6)	-
	3+	1 (0.1)	-	21 (2.8)	-	18 (1.1)	-

^1^Age at diagnosis, number of affected relatives and number of melanoma primaries were not available for seven Spanish cases.

^2^Data on family history were not reported for controls as this information is not relevant to this analysis, and number of primary melanomas was not reported as no controls had melanoma at recruitment to the study.

A total of 2929 melanoma cases and 1084 controls were screened for mutations in *CDKN2A* exons 1α, 1β, 2 and 3 and *CDK4* exon 2. No variants were identified in *CDK4* exon 2. Of the 58 *CDKN2A* variants identified in this study (Additional file 2) 33 were deemed very likely to be pathogenic mutations (Table 2 and Additional file 3). No pathogenic variants were identified in the control samples. Twelve different pathogenic variants were identified in the Australian cases (14 carriers of pathogenic mutations out of 596 cases in total), 11 in the Spanish cases (19 out of 747) and 17 in the UK cases (31 out of 1586) (Table 2).

**Table 2:** **Pathogenic** ***CDKN2A*** **variants identified in proband cases in Australia, Spain and the United Kingdom**

Gene element	Variant				Country		
	p16 nucleotide	p16 protein	p14 nucleotide	p14 protein	Australia	Spain	United Kingdom
5′UTR	c.-34G > T	r.-34_-32 > p.M1	_	_	1	1	1
Exon 1α	c.9_32del24	p.A4_10Edel7	_	_	.	.	3
	c.32_33ins9_32	p.M1_S8dup	_	_	1	.	4
	c.52_57dup6	p.20T_21Adup	_	_	1	.	1
	c.68G > A	p.G23D	_	_	.	.	1
	c.71G > C	p.R24P	_	_	.	.	1
	c.95T > C	p.L32P	_	_	1	.	1
	c.104G > A	p.G35E	_	_	.	1	.
	c.104G > C	p.G35A	_	_	.	.	1
	c.106delG	p.A36RfsX17	_	_	.	1	.
	c.113C > G	p.P38R	_	_	.	.	1
Exon 1β	_	_	c.45_60dup	p.V22PfsX46	.	1	.
	_	_	c.81C > G	p.I27M	1	.	.
	_	_	c.102G > A	p.W34X	1	.	.
	_	_	c.193G > A	p.G65S	1	.	.
	_	_	c.193G > C	p.G65R	1	.	.
Exon 2	c.159G > C	p.M53I	c.202G > C	p.D68H	.	.	5
	c.176T > G	p.V59G	c.219T > G	p.S73R	.	3	.
	c.188T > C	p.L63P	c.231T > C	p.A77A	.	.	2
	c.194T > C	p.L65P	c.237T > C	p.A79A	1	1	.
	c.202_203GC > TT	p.A68L	c.245_246GC > TT	p.R82L	1	.	.
	c.206A > G	p.E69G	c.249A > G	p.G83G	3	.	.
	c.212A > C	p.N71T	c.255A > C	p.Q85H	.	.	1
	c.228_246del19	p.L77TfsX62	c.271_289del19	p.R90VfsX75	.	.	2
	c.240_253del14	p.81PfsX56	c.92_105del14	p.95TfsX56	.	.	1
	c.251A > C	p.D84A	c.294A > C	p.R84R	.	.	1
	c.259C > T	p.R87W	c.302C > T	p.P101L	.	2	.
	c.301G > T	p.G101W	c.344G > T	p.R115L	.	5	.
	c.331G > A	p.G111S	c.374G > A	p.G125E	.	.	1
	c.358delG	p.E120SfsX26	_	_	.	1	.
	c.370C > T	p.R124C	_	_	.	2	.
	c.379G > T	p.A127S	_	_	.	1	.
Intron 2	c.458-105A > G	p.156_157del	_	_	1	.	4
Total					14	19	31

The prevalence of pathogenic mutations observed for melanoma cases at each centre was similar, with an overall frequency of 2.2% (95% CI: 1.7, 2.7%) for the three populations investigated with no evidence of any differences across the populations (chi-square test, p = 0.63) (Table 3).

**Table 3:** **Number and percentage of individuals for whom pathogenic and low penetrance** ***CDKN2A*** **variants were identified**

Variant			Australia	Spain ^1^	UK	Overall
*CDKN2A* high penetrance mutation	present	cases	14 (2.3)	19 (2.5)	31 (2.0)	64 (2.2)
		controls	0 (0)	0 (0)	0 (0)	0 (0)
	wild type	cases	582 (97.7)	728 (97.5)	1555 (98.0)	2865 (97.8)
		controls	476 (100)	109 (100)	499 (100)	1084 (100)
A148T variant	present	cases	48 (8.1)	63 (8.4)	110 (6.9)	221 (7.6)
		controls	18 (3.8)	6 (5.6)	32 (6.4)	56 (5.2)
	wild type	cases	548 (91.9)	684 (91.6)	1476 (93.1)	2708 (92.4)
		controls	458 (96.2)	102 (94.4)	467 (93.6)	1027 (94.8)
c.*29C > G variant	present	cases	163 (27.4)		425 (26.9)	588 (27.0)
		controls	125 (26.7)		133 (27.4)	258 (26.8)
	wild type	cases	433 (72.6)		1155 (73.1)	1588 (73.0)
		controls	351 (73.7)		352 (72.6)	703 (73.2)
c.*69C > T variant	present	cases	111 (18.6)		291 (18.4)	402 (18.5)
		controls	485 (81.4)		1289 (81.6)	1774 (81.5)
	wild type	cases	78 (16.4)		98 (20.2)	176 (18.3)
		controls	398 (83.6)		387 (79.8)	785 (81.7)

^1^There were no c.*29C > G or c.*69C > T variant data available for the Spanish sample.

The prevalence of pathogenic mutations increased with extent of family history (defined as the number of affected first or second degree relatives) for each of the three populations, rising, in the combined dataset, from 1.5% of cases for those without a family history to 5.2% for those with one affected relative, and to 25.5% for those with two or more affected relatives (Table 4). In Spain and the UK (combined), there was a corresponding increase with the number of lifetime melanoma primaries rising from 1.3% for cases with only a single primary to 6.5% for cases with two primaries and 29.3% for three or more. In Australia, where the eligibility criteria excluded those with a previous invasive melanoma, 2.4% of cases with a single primary at diagnosis had a mutation while none of the 15 cases with MPM (who presented with synchronous primary melanomas or a previous *in situ* primary melanoma) had a mutation (Table 4).

**Table 4:** **Association of family history and number of primaries with prevalence of** ***CDKN2A*** **mutations**

	Australia			Spain			UK				Total
Predictor	No./total cases ^1^	%	OR ^2^ (95% CI)	No./total cases	%	OR (95% CI)	No./total cases	%	OR (95% CI)	%	Multivariate OR ^3^
**Family history** ^**4**^											
0	7/500	1.4	1.0	13/696	1.9	1.0	20/1470	1.4	1.0	1.5	1.0
1	4/76	5.3	3.9 (1.1, 13.7)	3/35	8.6	4.9 (1.3, 18.2)	4/101	4.0	3.0 (1.0, 8.9)	5.2	3.8 (1.9, 7.5)
2+	3/20	15.0	12.4 (3.0, 52.3)	3/16	18.8	12.1 (3.1, 47.7)	7/15	46.7	63.4 (21.0, 19.8)	25.5	23.2 (11.3, 47.6)
**No. of primaries** ^**5**^											
1	14/581	2.4	n/a	8/644	1.2	1.0	20/1495	1.4	1.0	1.3	1.0
2	0/14	0		6/80	7.5	6.5 (2.2, 19.1)	4/73	5.5	4.3 (1.4, 12.9)	6.5	5.4 (2.5, 11.6)
3+	0/1	0		5/23	21.7	22.1 (6.6, 74.2)	7/18	38.9	46.9 (16.5, 133.5)	29.3	32.4 (14.7, 71.2)

^1^Number of cases with a pathogenic *CDKN2A* mutation/the total number of cases.

^2^Unadjusted odds ratio.

^3^“Family history” analysis ORs adjusted for presence of MPM, age at diagnosis and country; “Number of primaries” analysis adjusted for family history, sex, age at diagnosis and country.

^4^Number of first or second degree relatives with melanoma.

^5^Number of primary melanomas diagnosed in the case. Few MPMs were observed for the Australian cases due to the study design.

Compared with cases without a family history, cases with one affected first or second degree relative had a three-fold increased odds of carrying a mutation (OR 3.8; 95% CI: 1.9, 7.5), after adjusting for presence of multiple primaries, age and country, and for those with two or more affected relatives the odds of carrying a mutation was further increased (OR 23.2; 95% CI: 11.3, 47.6). The same trend was apparent for each of the three countries (Table 4).

For Spain and the UK, there was a significant positive association between number of primary melanomas identified in the case and the likelihood of carrying a pathogenic mutation (Table 4). Overall, compared with cases with a single primary melanoma, cases with two primary melanomas had a 5-fold increased odds of carrying a mutation (OR 5.4; 95% CI: 2.5, 11.6), after adjusting for family history, age and country; and for those with three or more primary melanomas the odds of carrying a mutation were much higher (OR 32.4; 95% CI: 14.7, 71.2).

The prevalence of mutations also increased for cases with multiple risk factors (family history plus multiple primaries). For instance, in Spain, mutation prevalence was 8.7% for MPM without a family history, 7.1% for cases with a family history but only one primary, and 17.4% for cases with MPM and a family history (data not shown). A similar pattern was seen for UK cases (7.5%, 5.7% and 45.4%) (data not shown). Table 5 shows, for the 3 countries combined, the prevalence of *CDKN2A* mutations, and the OR for finding a mutation for combinations of risk factors compared with the reference category of no family history and a single primary. For persons with two or more affected relatives and a personal history of three or more primary melanomas, the mutation prevalence was 60% and the OR for finding a mutation estimated as 146.6 (95% CI: 23.1, 928.1).

**Table 5:** **Cross-tabulation of mutation prevalence and odds ratios, according to family history and multiple primaries**

	No family history	One affected relative	Two or more affected relatives
**Prevalence of CDKN2A pathogenic mutation**			
One primary only	1.1% (27/2495)	3.8% (7/185)	20.0% (8/40)
Two primaries	3.6% (5/140)	14.3% (3/21)	33.3% (2/6)
Three or more primaries	26.7% (8/30)	20.0% (1/5)	60.0% (3/5)
**Odds ratios** ^**1**^ **and 95% confidence intervals**			
One primary only	1 (reference)	3.3 (1.4, 7.7)	18.9 (7.8, 45.5)
Two primaries	3.5 (1.3, 9.3)	13.1 (3.6, 47.8)	42.7 (7.5, 244.0)
Three or more primaries	33.1 (13.5, 81.5)	23.8 (2.6, 221.2)	146.6 (23.1, 928.1)

^1^Multivariate ORs adjusted for age at diagnosis and country.

Estimation of the expected numbers of cancers based on a multivariate model considering family history, the presence of multiple primaries, age, and sex, but unadjusted for country of recruitment, showed that this model predicted the number of observed carriers well (22.6 expected for Spain (observed = 19), 19.9 expected for the UK (observed = 22) and 12.5 expected for Australia (14 observed)) indicating that the predictors of having a mutation are consistent across populations independently of the baseline incidence rate or the precise method of ascertainment.

In Spain, there was a strong association between age at diagnosis and presence of a mutation, with carriers of a pathogenic mutation being diagnosed at age 35.9 years on average compared with 50.1 years for the cases not found to carry a pathogenic mutation in *CDKN2A* (p = 0.0002, data not shown). There was no similar association in the Australian (33.9 years vs. 32.7 years, respectively; p = 0.27) and UK samples (51.0 years vs. 53.8 years, respectively; p = 0.26), but the limited age range of the Australian study precluded further analysis. Within the Australian and UK samples, there was no evidence that mutation prevalence differed by gender (data not shown) but for Spain, 3.9% of male cases were carriers compared with 1.6% of female cases (p = 0.06 Fisher’s exact test).

The frequency of pathogenic mutations was similar for cases with melanomas at different body sites (head and neck; trunk; limbs; other) for Australia and Spain. For the UK, there was some evidence that cases with tumours on the trunk were more likely to carry a mutation than cases with tumours at other sites (Fisher’s exact test p = 0.04, data not shown). There was also some weak evidence that thinner tumours were associated with *CDKN2A* mutations for the UK samples (p = 0.05, data not shown), but not for the Australian or Spanish samples. For the UK, 3.3% of tumours with Breslow ≤1 mm had *CDKN2A* mutations, compared with 1.2% of those >1 mm.

### Analysis of lower penetrance *CDKN2A*variants

The low penetrance variant p.A148T was observed at an overall frequency of 7.6% for melanoma cases, and 5.2% for controls (p = 0.008) (Table 3) (p = 0.17 and 0.57 for homogeneity of prevalence among controls and cases, respectively). Crude ORs for risk of melanoma for the three countries were; Australia: 2.2 (95% CI: 1.3, 3.9); Spain: 1.6 (95% CI: 0.7, 3.7); and UK: 1.1 (95% CI: 0.7, 1.6). Analysis of all studies together suggested that presence of a variant increased melanoma risk (OR 1.5; 95% CI: 1.1, 2.0) (p = 0.009, data not shown). As the Australian cases were all <40 years of age, we examined whether the positive finding for Australia related to an effect of age at onset. Analysis from the UK supported this suggestion with ORs of 1.6 (95% CI: 0.7, 3.7) and 0.9 (95% CI: 0.6, 1.5) within the age groups ≤50 and >50 years respectively, with evidence of different effect sizes (p = 0.001). Conversely, the Spanish data suggested a stronger association for older onset cases: crude ORs were 0.9 (95% CI: 0.3, 2.4) for ≤50 years and 4.8 (95% CI: 0.6, 36.1) for >50 years, but the evidence for a difference was weak, p = 0.5. The carriage of the p.A148T variant was not associated with MPM (p = 0.14) or family history (p = 0.60).

The 3′ UTR variant c.*29C > G was observed at an overall frequency of 27.0% for cases and 26.8% for controls (Table 3). The 3′UTR variants were not analyzed for the Spanish sample, as they were located outside the limits of the SSCP screen employed. Crude ORs for risk of melanoma for Australia and the UK were 1.06 (95% CI: 0.81, 1.39) and 0.97 (95% CI: 0.77, 1.22) respectively. The 3′ UTR variant c.*69C > T was observed at an overall frequency of 18.5% for cases and 18.3% for controls. Crude ORs for risk of melanoma for Australia and the UK were 1.17 (95% CI: 0.85, 1.61) and 0.89 (95% CI: 0.69, 1.15) respectively.

## Discussion

We report a large study aimed at characterizing the prevalence and predictors of pathogenic *CDKN2A* mutations for melanoma cases. Its strengths include its size and participant recruitment at very different latitudes so that the effect of ambient sun exposure might be inferred. Its main weakness is that differences in case ascertainment for the three studies may have obscured real differences in mutation frequency between populations.

We found that population-based melanoma cases recruited at different latitudes had very similar probabilities of carrying a pathogenic mutation of the *CDKN2A* gene of 2.0-2.5%. This finding is consistent with that of the international, population-based GEM study [12]. These findings do not support our hypothesis that the prevalence of germline mutations would be higher for melanoma cases in a country with lower levels of sun exposure (UK) than for one in which melanoma cases with similar ethnicity (derived largely from UK immi gration) live with much higher levels of sun exposure (Australia).

We do not think that differences in mutation detection methodology are likely to have obscured any differences in mutation prevalence between countries, as SSCP, DHPLC and direct re-sequencing have been shown to have similar sensitivity and specificity [19]. The classification of variants as pathogenic or non-pathogenic is also vital for a study of this type [27]. Effects on protein function have not been investigated for many *CDKN2A* variants identified by this and other studies. We have used a range of evidence to classify variants where there are no available functional data. We consider that this approach gives an accurate prediction of the likely pathogenicity associated with each variant; our classification of variants almost exactly matches that determined using a Bayesian approach to classification [28]. The only exception was the variant A127S; classified as ‘probably neutral’ using the Baysian model, but presented here as pathogenic, because of reported evidence of possible functional impairment [29].

The overall frequency of pathogenic mutations determined by this study (2.2%, 64/2929) is slightly higher than reported by the GEM study (1.8%, 65/3613) [12] (Fisher’s Exact p = 0.28 for difference). This is possibly due to the inclusion of *CDKN2A* exon 1β in this mutation screen, and the extension of the exon 3 PCR to cover the common, pathogenic, intron 2 variant c.458-105A > G [30], neither of which were included in the GEM screen [12] but which account for 10 carriers (0.34%) in our sample. Our estimate is lower than a recent report from Greece, where 5% of cases had mutations [31], but is equivalent to the 2% frequency of *CDKN2A* mutations identified by an Icelandic study [32]. In both the Greek and Icelandic studies, specific founder mutations dominated the spectrum of mutations observed. Clearly, founder population effects can influence the overall picture; in this study a much more even distribution of *CDKN2A* mutations was observed, with no single variant predominant across the three populations.

We found the prevalence of pathogenic mutations in *CDKN2A* was very low (~1%) for cases without a family history or MPM. The prevalence increased with the presence of either family history or MPM (~8%), and was highest for cases with both a family history and MPM (~25%). This confirms that the most important predictors of germline *CDKN2A* mutations in melanoma cases unselected for family history of the disease are the same as for those from multiple-case families: namely the presence of multiple primaries and the strength of the family history [7].

Earlier age at onset has previously been shown to be associated with an increased prevalence of *CDKN2A* mutation [11, 12]. Our analysis detected an age effect only for Spanish samples, as was previously reported for Spanish MPM cases [13]. The observation that for the Spanish population pathogenic mutations appear to be slightly more frequent for males than females is also consistent with previous studies [12, 13] in Spain and the GEM study, which recruited from Europe, the USA and Australia. We did not observe this sex difference for the UK and Australia and its significance remains unclear.

Melanoma cases with thinner tumours appeared to have an increased prevalence of *CDKN2A* mutations in the UK sample set in which there was a greater proportion of thicker tumours than in the other two studies (in part because of an ascertainment choice). It is possible that this may be a result of people with a family history of melanoma having their skin checked more regularly resulting in their melanoma being detected at an earlier stage, and is consistent with observations that subsequent melanomas are thinner than first melanomas [33]; but it may also have been a chance finding. The GEM study also reported a slightly higher prevalence of *CDKN2A* mutations in thinner tumours (p = 0.07) [12]. In the UK there was a suggestion that an increased proportion of individuals with tumours on the trunk carried mutations of *CDKN2A.* This could indicate that *CDKN2A* mutation plays a greater role in tumour development at intermittently sun exposed body sites. Because of limited sample size, the data from Australia and Spain do not add to this interpretation.

This is the first large series of melanoma cases to report pathogenic mutations in exon 1β, which uniquely affects p14ARF, for cases not selected because of their family history of melanoma or because they have multiple melanomas. In the Australian sample one case carried a nonsense mutation p.W34X, and two cases had mutations that disrupted the splice acceptor site, a known mutation hotspot for this gene in familial melanoma.

We found no evidence that the common 3′ UTR variants, c.*29C > G and c.*69C > T were associated with melanoma susceptibility. Conflicting data have been published on the significance of the low penetrance variant p.A148T and melanoma risk [22, 23, 26]. We observed a significantly increased risk associated with the p.A148T variant in Australia, and the combined estimate of the effect of the p.A148T variant across populations was 1.45 (1.06, 1.98), with no evidence of heterogeneity across populations (p = 0.56). Further, as all Australian cases had early onset disease, we divided the other countries’ data by age (< = 50, vs. >50 years) and found a significant difference in the OR by age for the UK sample, suggesting a stronger effect at younger age (p = 0.001). Therefore an effect of the p.A148T variant on melanoma risk, perhaps greater in younger people, is plausible from our data.

## Conclusions

Our data suggest that in most clinical settings, testing for *CDKN2A* mutations is very unlikely to yield a positive result (<2% probability) except for melanoma cases with a strong family history of melanoma, those who have had three or more primary tumours, and individuals with more than one primary tumour and who also have other affected family members, where the overall prevalence of mutations is estimated to be approximately 25%, 30% and 24% respectively. This provides additional evidence supporting present advice to clinical geneticists on genetic testing for melanoma [34].

## Electronic supplementary material

Additional file 1: **Flow chart for variant classification.** Classification of identified *CDKN2A* variants using the following criteria: having functional evidence of impaired protein function; predicted to result in protein truncation; previously reported co-segregation with disease in large melanoma families; and prediction of a strong deleterious effect on *CDKN2A* protein by bioinformatic criteria. (PDF 227 KB)

Additional file 2: ***CDKN2A*** **variants identified in cases and controls recruited In Australia, Spain and the United Kingdom.** Full list of all variants identified in this study. (XLSX 51 KB)

Additional file 3: **Evidence for pathogenicity of identified point mutations In CDKN2A.** Bioinformatic, segregation, and functional evidence used in the classification of *CDKN2A* variants. (XLSX 43 KB)

## Abbreviations

UV: Ultraviolet
OR: Odds ratio
UK: United Kingdom
*CDKN2A*: cyclin-dependent kinase inhibitor 2A.
