# Supplementary figures and images for: Prevalence and predictors of germline CDKN2A mutations for melanoma cases from Australia, Spain and the United Kingdom
Source: Hered Cancer Clin Pract. 2014 Nov 20;12(1):20. doi: 10.1186/1897-4287-12-20 (PMC4361137; doi:10.1186/1897-4287-12-20)

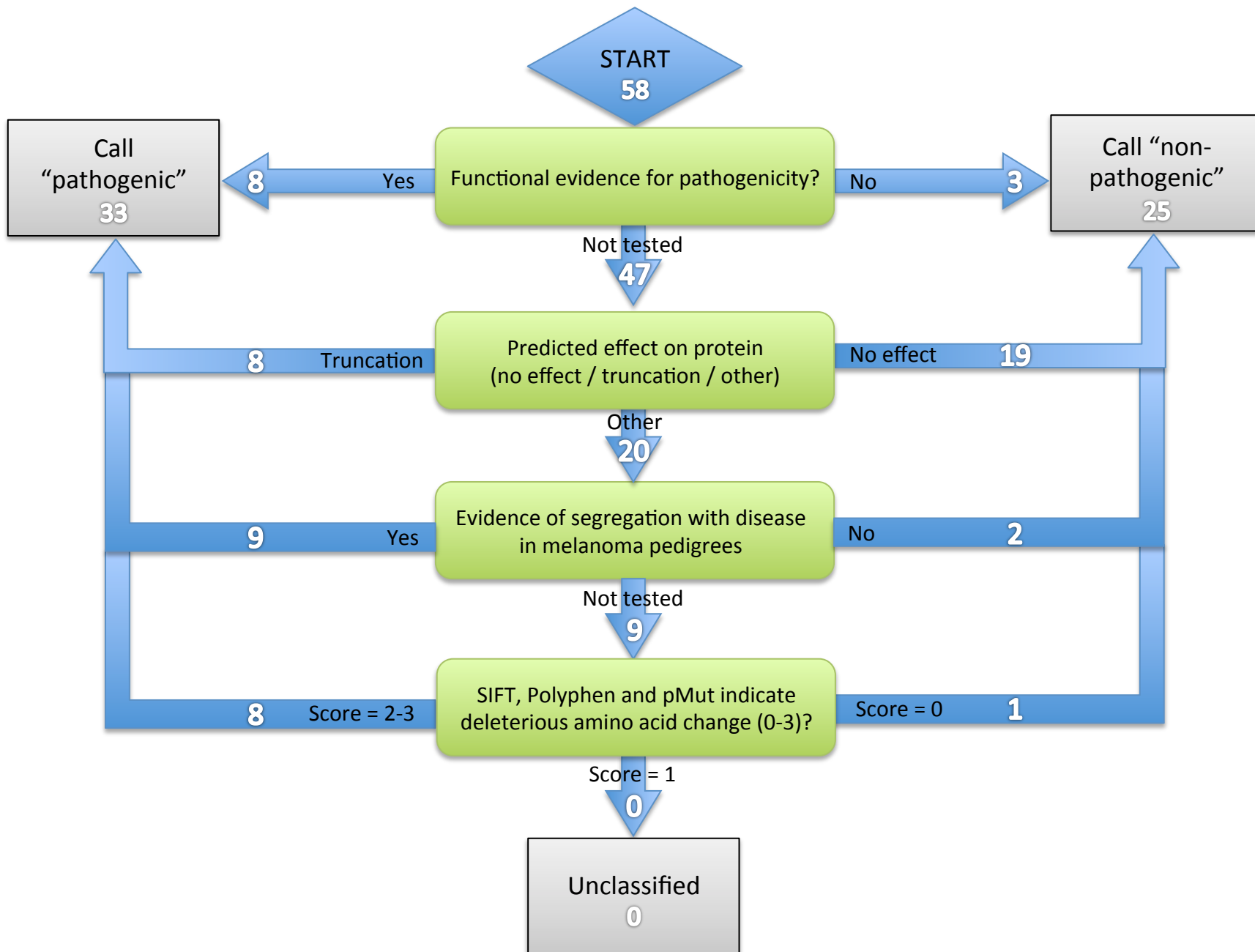

Supplement: Supplementary file 1 — Additional file 1: Flow chart for variant classification. Classification of identified CDKN2A variants using the following criteria: having functional evidence of impaired protein function; predicted to result in protein truncation; previously reported co-segregation with disease in large melanoma families; and prediction of a strong deleterious effect on CDKN2A protein by bioinformatic criteria. (PDF 227 KB) [file 13053_2014_472_MOESM1_ESM.pdf]
